# Integrating trait‐based empirical and modeling research to improve ecological restoration

**DOI:** 10.1002/ece3.4043

**Published:** 2018-05-29

**Authors:** Sebastian Fiedler, Michael P. Perring, Britta Tietjen

**Affiliations:** ^1^ Biodiversity/Theoretical Ecology Institute of Biology Freie Universität Berlin Berlin Germany; ^2^ Berlin‐Brandenburg Institute of Advanced Biodiversity Research (BBIB) Berlin Germany; ^3^ Forest and Nature Lab Ghent University Gontrode‐Melle Belgium; ^4^ Ecosystem Restoration and Intervention Ecology Research Group School of Biological Sciences The University of Western Australia Crawley WA Australia

**Keywords:** ecosystem functions, ecosystem services, Mediterranean‐type ecosystem, multifunctional ecosystems, resilience, simulation models

## Abstract

A global ecological restoration agenda has led to ambitious programs in environmental policy to mitigate declines in biodiversity and ecosystem services. Current restoration programs can incompletely return desired ecosystem service levels, while resilience of restored ecosystems to future threats is unknown. It is therefore essential to advance understanding and better utilize knowledge from ecological literature in restoration approaches. We identified an incomplete linkage between global change ecology, ecosystem function research, and restoration ecology. This gap impedes a full understanding of the interactive effects of changing environmental factors on the long‐term provision of ecosystem functions and a quantification of trade‐offs and synergies among multiple services. Approaches that account for the effects of multiple changing factors on the composition of plant traits and their direct and indirect impact on the provision of ecosystem functions and services can close this gap. However, studies on this multilayered relationship are currently missing. We therefore propose an integrated restoration agenda complementing trait‐based empirical studies with simulation modeling. We introduce an ongoing case study to demonstrate how this framework could allow systematic assessment of the impacts of interacting environmental factors on long‐term service provisioning. Our proposed agenda will benefit restoration programs by suggesting plant species compositions with specific traits that maximize the supply of multiple ecosystem services in the long term. Once the suggested compositions have been implemented in actual restoration projects, these assemblages should be monitored to assess whether they are resilient as well as to improve model parameterization. Additionally, the integration of empirical and simulation modeling research can improve global outcomes by raising the awareness of which restoration goals can be achieved, due to the quantification of trade‐offs and synergies among ecosystem services under a wide range of environmental conditions.

## 1 INTRODUCTION

The provision of ecosystem services that people rely on for their well‐being is declining worldwide, a decline which is likely to continue in light of multiple global changes (e.g., land use, biotic invasion, and climate; MEA, 2005). Improving the long‐term supply of ecosystem services necessitates strategies to assist degraded, damaged, transformed, or even destroyed ecosystems (Bullock, Aronson, Newton, Pywell, & Rey‐Benayas, 2011). Ecological restoration at regional and landscape scales is increasingly touted as being one such viable strategy, and this recognition has recently led to a global agenda to fully commit to restoration (Rey Benayas, Newton, Diaz, & Bullock, 2009; SER, 2004; Shackelford et al., 2013; Suding et al., 2015). Here, we suggest that current and future restoration approaches might not achieve a goal of resilient (i.e., the ability of ecosystems to absorb changes of state variables, driving variables, and parameters and still persist after disturbances; Holling, 1973), multifunctional ecosystems due to a lack of knowledge about trade‐offs among multiple ecosystem services (Bennett, Peterson, & Gordon, 2009) as well as the effect of multiple changing environmental factors on services. We propose a framework that integrates simulation modeling and experimental approaches to address this critical knowledge gap.

Arguments have been advanced that incorporating approaches focusing on plant functional traits—measurable properties of an individual plant or plant species, which can be compared across individuals and plant species, such as plant height, the specific leaf area, or specific root length (Bardgett, Mommer, & de Vries, 2014; McGill, Enquist, Weiher, & Westoby, 2006; Violle et al., 2007)—can improve ecological restoration outcomes toward ecosystem service delivery (Funk, Cleland, Suding, & Zavaleta, 2008; Laughlin, 2014a; Perring et al., 2015). These measurable traits have been found to be linked to ecosystem processes that drive the transfer of energy and/or materials, such as nutrients and water, over time and space—so called ecosystem functions—(Lavorel & Garnier, 2002), which provide the base for the provision of ecosystem services (Daily, 1997).

Until now, most trait‐based approaches have studied the effect of plant traits on only a single ecosystem function or service and thereby a priori neglected possible trade‐offs among multiple functions/services (e.g., Ruiz‐Benito et al., 2014; Simpson et al., 2016; further examples in Tables S1 and S2). These trade‐offs are potentially very important for service delivery. For instance, the plant trait “leaf area per unit ground surface area” (LAI) is positively linked to photosynthesis (Gratani, Varone, Ricotta, & Catoni, 2013), and species with high LAI may therefore be chosen to reach a goal of increased carbon sequestration. However, higher leaf area per unit dry mass (SLA, specific leaf area), which is positively correlated to LAI (Pierce, Running, & Walker, 1994), might at the same time negatively impact soil water content due to decreased water use efficiency (Medrano, Flexas, & Galmés, 2009), which might result in a trade‐off between carbon sequestration and water retention.

In addition, individual traits may not only be linked to individual functions (Medrano et al., 2009). Instead, multiple traits can influence one function, and multiple functions can be influenced by a single trait (de Bello et al., 2010 and examples in Tables S1 and S2). As such, it is difficult to suggest traits that vary orthogonally, that is, that independently represent different functions. Although there is some evidence to suggest that there are orthogonal axes that determine plant strategies (e.g., the leaf‐height‐seed strategy scheme of Westoby, 1998), and there are thus a few traits that are a good description of plant responses to environmental change, subsequent research has shown correlations among even these axes (Garnier, Bellmann, Navas, Roumet, & Laurent, 2004; Lavergne, Garnier, & Debussche, 2003). In addition, there may also be other axes to consider (Laughlin, 2014b) and the fact that traits that respond to environmental change may have different effects on ecosystem functioning (Suding et al., 2008). As such, it will be valuable for both restoration and fundamental ecological understanding to continue to identify traits important to ecosystem service delivery, quantify covariation among traits across scales, and to assess whether there is environmental context dependency in this covariation (Funk et al., 2017; Garnier, Navas, & Grigulis, 2016; Vilà‐Cabrera, Martínez‐Vilalta, & Retana, 2015).

The strength and direction of the links between traits, functions, and services also need to be assessed for multiple environmental change settings, such as different combinations of land use, biotic invasion, and climate. This will enable plant trait compositions to be identified that are likely resilient to multiple factors, given traits and function maintain their association, thus, allowing continued provision of multiple ecosystem functions and services. So far, the effects of single environmental factors on plant traits and ecosystem functions are well investigated (e.g., Cochrane, Hoyle, Yates, Wood, & Nicotra, 2015; LeRoy, Wymore, Davis, & Marks, 2014; Prieto et al., 2015), but less attention has been given to the simultaneous effects of multiple changing factors (see Table S2). This is an important knowledge gap, as the overall effect of multiple factors may not be a simple sum of the individual effects (so called additive effects). Instead, the overall effect might result from the interaction of multiple changing environmental factors that cannot be predicted by the sum of the individual effects (so called nonadditive or interactive effects). For instance, nitrogen fertilization can increase the negative effect of drought on biomass production due to increased evaporative demands (Meyer‐Grünefeldt, Friedrich, Klotz, Von Oheimb, & Härdtle, 2015). Accordingly, there might be nonadditive effects of nitrogen deposition and increasing aridity on carbon sequestration, emphasizing the importance of accounting for simultaneous impacts of multiple changing factors on the provision of ecosystem services. Most trait‐based studies primarily focused on single environmental factors, and studies on simultaneous changes and thereby considering interacting effects of more than two changing environmental factors on ecosystem functions and services via plant traits are rare (e.g., Ashbacher & Cleland, 2015; Pérez‐Camacho et al., 2012; see Table S2).

In addition to direct effects of changed factors, the indirect effects of these factors via changes in plant trait composition hamper the assessment of changes in ecosystem functions. For example, an increase in temperature directly impacts nutrient supply by the increased rate of litter decomposition (Rustad et al., 2001). As temperature might also impact plant species composition and thus litter quality, this could additionally indirectly impact decomposition rates and nutrient supply (LeRoy et al., 2014; Sariyildiz, Anderson, & Kucuk, 2005). Until now, there are in fact numerous short‐term studies that particularly evaluated the direct effects of environmental factors on plant traits as well as on ecosystem functions (see Table S2). However, only a few studies have taken into account the potentially important indirect effects of environmental change on ecosystem functioning via changing plant traits (e.g., Godoy, Castro‐Díez, Van Logtestijn, Cornelissen, & Valladares, 2010; Valera‐Burgos, Zunzunegui, & Díaz‐Barradas, 2013).

**Figure 1:**
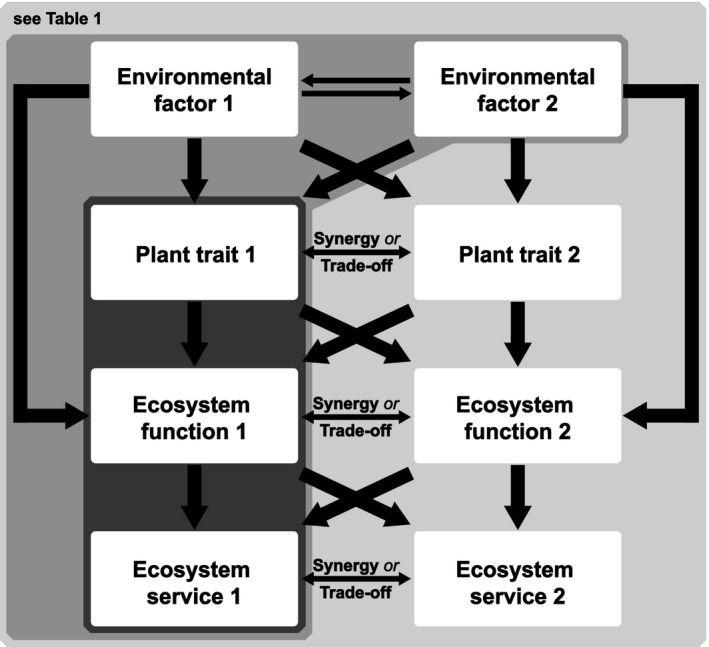
Components (boxes) and relationships (arrows) needed to assess the resilient provision of multiple ecosystem services. Based on literature for Mediterranean‐type ecosystems, trait‐based studies can be categorized as those that consider the effect of plant traits on (single) ecosystem functions and services (dark gray area, see Table S1) and as those that consider the effects of changing environmental factors on single plant traits and/or on single ecosystem functions and services (medium gray area, see Table S2). Table 1 (light gray area) explores the integration of simulation modeling and empirical approaches to tackle the research gaps identified by this framework

In summary, most trait‐based studies do not explicitly account for the full path from changing environmental factors via plant traits to ecosystem functions and services as given in Figure 1. Instead, they focus on only single links in the pathway and neglect interactions among environmental factors themselves, and between changing environments, plant traits, and functions. Thus, it is currently not clear to what extent the goal of restoring resilient multiple ecosystem services can be successfully achieved. A major reason for this knowledge gap might be that empirical studies often allow only for a limited complexity of the experimental design and short‐time scales of assessment, due to restricted financial, spatial, or other resources. Therefore, a full factorial design, in which all plant trait combinations are integrated and changes in various environmental factors are evaluated to assess the long‐term supply of various ecosystem functions and services, is normally not feasible. Process‐based ecological simulation models that describe a simplified representation of an ecosystem, including its components such as individual plants and processes such as plant growth, and that explicitly account for plant traits could close the gap. However, such models depend on field data for model input (e.g., time series of weather conditions), parameterization (e.g., trait measurements such as specific leaf area) and validation of the model output (e.g., aboveground biomass). Here, we suggest that to fully realize the potential of trait‐based approaches, empirical and simulation modeling research agendas need integrating.

In the following, we outline a stepwise research agenda that integrates empirical research and simulation modeling to better understand environmental change and plant trait effects on ecosystem services. We argue that implementing this agenda will aid practitioners and scientists in their aim of reinstating and maintaining ecosystem services on degraded land. Although we illustrate our research agenda with reference to Mediterranean‐type ecosystems, our arguments pertain to furthering ecological restoration globally.

## 2 THE WAY FORWARD: INTEGRATING TRAIT‐BASED EMPIRICAL AND SIMULATION MODELING RESEARCH

Achieving a resilient supply of ecosystem services toward future environmental change requires integrative approaches that combine the knowledge gained from empirical studies with process‐ and trait‐based simulation models. Such integrative approaches, however, have been generally missing until now. Ideally, the coupled approach should be initiated at the same time to identify synergies between empirical and modeling approaches at the earliest opportunity: for example (1) what are the joint research questions, (2) how can modeling and empirical research complement each other, (3) what components and processes of the system should be included to answer these questions, and (4) what data should be measured for model parameterization and validation.

**Figure 2:**
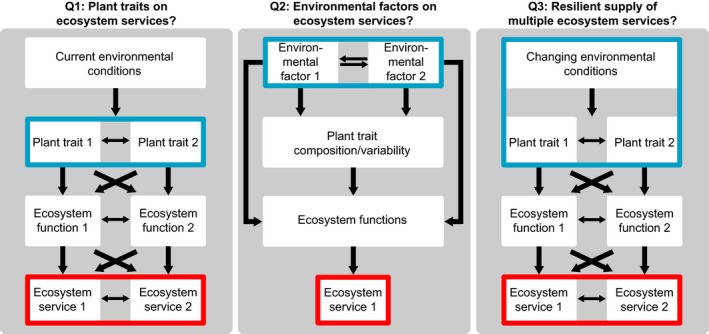
Schematic overview of potential research questions (Q1–Q3) that could be answered with the coupled approach. Boxes and arrows indicate which relationships among environmental conditions, plant traits, ecosystem functions, and services are addressed in each question. The blue boxes indicate the factor(s) that are systematically changed to answer the questions Q1–Q3, whereas the red boxes indicate the respective output(s)

To achieve the goal of multifunctional and resilient ecosystems, we suggest the following fundamental and applied research questions need tackling (Figure 2):

1. Which relationships among ecosystem services result from reasonable plant trait compositions under current environmental conditions?
2. What are the indirect and direct impacts of changing environmental factors on ecosystem functioning? And which simultaneous effects of multiple changing environmental factors on ecosystem functioning and service provisioning are nonadditive and why?
3. Are there plant trait compositions that provide a resilient supply of multiple ecosystem services under global change?

Here, we briefly propose and describe three consecutive steps of a coupled agenda that describes how empirical and modeling research can be integrated to achieve the ultimate goal of multifunctional and resilient ecosystems (Table 1). We then elaborate these steps using an ongoing case study to illustrate the potential power of our approach.

**Table 1:** Framework of a coupled trait‐based empirical and simulation modeling approach to improve ecological restoration toward resilient and multifunctional ecosystems. Shown are three main consecutive steps stating the goal of each step, the actions needed in a simulation modeling approach, and the linkages to empirical approaches

Goal	Simulation modeling approach	Link to empirical approach
Step 1: Development of trait‐based simulation model		
Existence of fully coupled ecosystem model that links from traits to ecosystem services	Implementation of coupled vegetation, water and nutrient processes, and their linkage to plant traits	Model parameterization based on measured plant traits, climatological data, and soil properties
	Definition of ecosystem measures to quantify ecosystem services	Additional empirical experiments proposed during model development
Step 2: Model validation and testing		
Gain confidence in modeled outputs and understand their sensitivity to parameters	Simulation experiments that resemble the empirical experiments for model validation	Model validation based on measured fluxes and states
	Sensitivity analyses of parameters	Comparison of modeled and measured ecosystem services
Step 3: Simulation experiments of scenarios and restoration options		
Improve restoration outcomes by detecting species compositions providing multiple ecosystem services resilient to environmental change	Long‐term model simulations on multiple plant species compositions and changing environmental factors	Model assesses the same but also additional plant species combinations and treatments
	Evaluations of trade‐offs and synergies among ecosystem services	Model suggests improved species combinations that are then planted and monitored to inform future work
	Evaluation of additive and nonadditive effects of multiple environmental factors	

### 2.1 Step 1: Development of trait‐based simulation model

Empirical approaches can improve our understanding for mostly shorter‐term ecosystem dynamics and less complex experimental designs (e.g., question 1). Models can complement this by assessing more complex designs (e.g., question 2) as well as the long‐term success of restoration efforts (e.g., question 3). In order to answer more complex questions, a model should be developed that defines and simulates ecosystem stocks and fluxes that global changes influence and that underpin restoration goals in a coupled manner. Often validated models or processes already exist in the literature, and they only have to be adapted to the system studied (e.g., by including the effect of nutrient availability on plant growth) or newly linked (e.g., by coupling of vegetation, nutrient, and water processes). In answering our research questions, model components should include water, nutrient, and vegetation processes (respectively, e.g., infiltration, mineralization, and growth as a function of photosynthesis and respiration) and associated stocks (e.g., moisture at different soil depths, nutrient availability, and above‐ and belowground plant biomass). In addition, we need to incorporate explicitly plant traits that determine these dynamics, along with abiotic conditions. Incorporating traits in simulation models, rather than specific species, would also allow for assessing the whole variability range of a trait, both intra‐ and interspecifically. In addition, using plant traits with clear links to measured ecosystem functions and services is a prerequisite to better connect empirical and simulation modeling research. The specific empirical data required to feed into and assess simulations will depend upon the questions posed. We elaborate this in an example case study below and also highlight the challenges that require addressing to enable integration.

### 2.2 Step 2: Model validation and testing

The step of model validation and testing is a crucial step to gain full confidence of the model developed which should always be repeated once the model has been changed or before it will be applied to another system. Local (single changed parameters) or global sensitivity analyses (multiple changed parameters at once) of model outcomes may be performed to find sensitive parameters that should be parameterized with high precision as well as less sensitive parameters for which some uncertainty can be accepted (Reuter, Jopp, Breckling, Lange, & Weigmann, 2011; Ruget, Brisson, Delécolle, & Faivre, 2002). However, if a sensitive parameter is uncertain, this uncertainty should be propagated through model simulations to establish a full range of potential outcomes (e.g., via an uncertainty analysis, see e.g. Hopfe & Hensen, 2011). For model validation, simulated dynamics should be compared to measured dynamics that have not been used for model parameterization (e.g., biomass dynamics that have not been used to calculate the growth rate). Process validation can require custom‐made assumptions of model goodness (see e.g., Reuter et al., 2011; Sargent, 2013). If a stock cannot be validated, the description of the involved model processes might be adapted (see Step 1). Once the model is satisfactorily validated (Oreskes, Shrader‐Frechette, & Belitz, 1994), simulation experiments for answering the research questions can be performed (see Step 3).

### 2.3 Step 3: Simulation experiments of scenarios and restoration options

Model experiments do not only resemble empirical experiments for model validation (see Step 2). Calculated simulations can additionally complement shorter‐term empirical studies by evaluating a full factorial design of multiple changing environmental factors as well as plant species composition scenarios and by assessing potential long‐term effects. A simulation modeling approach allows modifying environmental changes singly, or together. A major challenge is that multiple changes occur simultaneously and there are an overwhelming number of relationships; this ability of models to simulate factors in a controlled manner allows investigating likely mechanisms behind ecosystem responses. One can also consider whether environmental change factors themselves interact and assess the outcome of such relationships. More and more complex scenarios (e.g., with more environmental changes, a greater number of ecosystem functions) can be efficiently analyzed with such a modeling approach. Indeed, Figure 1 only hints at the complexity of the situation—environmental factor 2 could have direct effects on ecosystem function 1, while there is the potential for more than two environmental factors to be changing. The outcome of the factorial experiments allows for a systematic assessment of trade‐offs and synergies among multiple ecosystem services. Direct and indirect effects, and additive and nonadditive interactions, of multiple changing environmental factors can also be evaluated. As a result, restoration scientists and practitioners can assess which trait compositions, if any, maximize the resilient supply of multiple ecosystem services in the face of simultaneous environmental changes. During this step, we can potentially generate better hypotheses of what will happen over time and across space outside of the empirically measured system, which can then be tested by additional empirical experiments. The outcome of such additional experiments can help to improve the development of the ecosystem model.

## 3 CASE STUDY—THE RIDGEFIELD RESTORATION EXPERIMENT

We exemplify our integrative agenda using an ongoing case study with focus on Mediterranean‐type ecosystems. Although these systems cover only about 2% of the global terrestrial area, they contain about 20% of all plant species with a high degree of endemism (Cowling, Rundel, Lamont, Arroyo, & Arianoutsou, 1996; Médail & Quézel, 1997). Long‐term extensive human activity has contributed to the high biodiversity in Mediterranean‐type ecosystems (Bugalho, Caldeira, Pereira, Aronson, & Pausas, 2011). However, altered and intensified anthropogenic land use during the last century combined with other factors of global change (e.g., biotic invasion, climate, nitrogen deposition, and atmospheric CO_2_) led to the contemporary threatening situation for their unique biodiversity (IPCC, 2013; Sala, 2000), making them global biodiversity hotspots (Myers, 1990). Worldwide, many Mediterranean‐type regions undergo a similar fate: deforestation, unsustainable agricultural and management practices, urbanization, and invasion by alien species are the major threats (Cowling et al., 1996). As a result of ecosystem degradation, ecosystem functions have altered. These changes lead to an increased fire hazard, decreased carbon sequestration, desertification, soil and water erosion, salinization, and nutrient losses (Hobbs, 1998; Vallejo, Aronson, Pausas, & Cortina, 2001). Ongoing and future alterations in global change factors have the potential to exacerbate degradation of Mediterranean‐type ecosystems, leading to a further decrease in their provision of ecosystem services (Mace, Norris, & Fitter, 2012; MEA, 2005; Sala, 2000). This requires plant communities that could be planted to restore Mediterranean‐type ecosystems with respect to their ecosystem service supply as well as their resilience to future threats.

**Figure 3:**
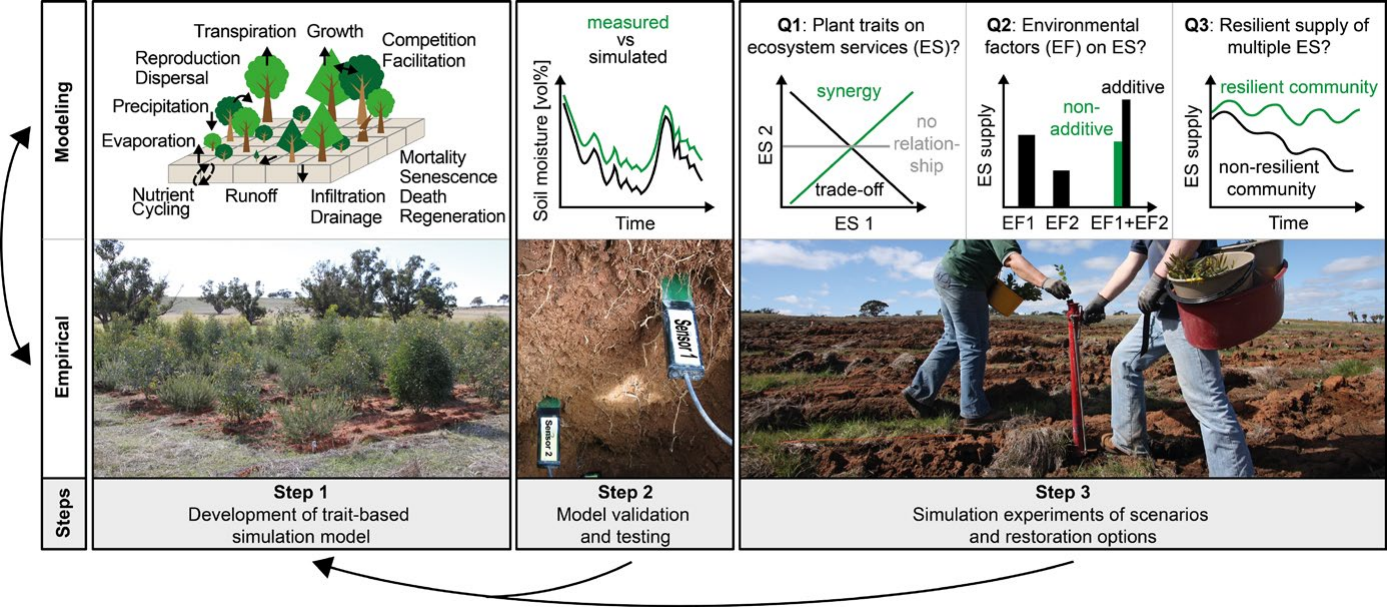
Steps of the coupled trait‐based simulation modeling (first row) and empirical approach (second row) in our case study. Step 1 shows a model that simulates the fate of individual plants by calculating soil water, nutrient, and plant processes in a spatially explicit landscape divided into grid cells (first row) as well as a picture showing a plot of the large‐scale restoration experiment in SW‐Australia, Ridgefield (second row, © Richard J. Hobbs, 2012). Step 2 exemplifies how to validate the model by a comparison of simulated and measured soil moisture dynamics (first row) that was measured with soil sensors in different soil depths in Ridgefield (second row). Step 3 shows how to assess the research questions as shown in Figure 2 (first row). The first question (Q1) compares the outcome of two ecosystem services at a certain point in time and assess the relationships among them (no relationship, synergy, or trade‐off). Additive and nonadditive effects of multiple environmental factors (Q2) are assessed through comparing the effects of single changes on the delivery of ecosystem services with the effects of combined changes. The third question (Q3) models initial plant trait compositions and asks which provide ecosystem services in a resilient manner over time. Those compositions can then be planted to aid restoration of degraded ecosystems (second row, © Cristina E. Ramalho, 2010). Importantly, these are monitored to assess whether supply of ecosystem services is resilient. Findings from both Step 2 and Step 3 can be used to further improve the simulation model, as indicated by the arrow returning to Step 1

To find these ideal plant communities, our approach is integrating a large‐scale field experiment in an agricultural landscape in South West Australia (the Ridgefield Experiment, Perring et al., 2012) with a simulation model. The model (currently under development) is being parameterized through measurements at the site, to eventually investigate the long‐term effects of functional diversity and multiple environmental factors on the supply of multiple ecosystem services, and trade‐offs and synergies among them. The intention of future modeling will be to close the knowledge gaps to further the research field of restoration ecology, for example, in terms of process knowledge, suitable trait combinations and transferability of site‐specific knowledge to other environmental conditions. In the following, we will describe the application of the three consecutive steps we argue are necessary to integrate simulation and empirical trait‐based research. This description highlights the actions and potential links between simulation modeling and the field experiment that each step involves in order to address our research questions (Figure 3).

### 3.1 Step 1: Development of trait‐based simulation model

In our coupled study, the Ridgefield experiment was set up in August 2010 (Perring et al., 2012), whereas the model development has started recently (Figure 3, Step 1).

Although various trait‐based simulation models of Mediterranean‐type ecosystems exist and have been used, for example, to assess the impact of climate and fire on vegetation composition or performance, none of these models can currently fully assist restoration efforts toward multifunctional and resilient ecosystems. For example, several model approaches neglect soil water and nutrient dynamics, as well as their feedbacks to vegetation dynamics (e.g., Esther et al., 2011; Moore & Noble, 1990; Pausas, 1999) and are therefore too simplified to assess the impact of global change. Other models explicitly consider water dynamics, but neglect nitrogen dynamics (e.g., Fyllas & Troumbis, 2009; Mouillot, Rambal, & Lavorel, 2001) and thus cannot account for the effects of nutrient deposition, for example, on invasive species or on ecosystem functions such as dissolved and particulate leaching and gaseous nutrient loss. In addition, these models are often rather conceptual and thus not thoroughly parameterized and validated against field data, which limits their suitability for applied restoration projects.

Therefore, we are developing a process‐based model that addresses the issues raised by linking processes for calculating water, nutrient, and vegetation dynamics.

#### 3.1.1 Model overview

We divided the total modeled landscape (25 by 25 m², reflecting a plot in Ridgefield) into grid cells (each cell: 5 by 5 m²) and different soil layers per cell. The size of the grid cells and the depth of the different layers depend on the site‐specific soil heterogeneity. Each layer is defined by soil traits characterizing the local prevalent soil texture. Individual plants are distributed over the landscape and are characterized by plant traits. The main simulated ecosystem stocks that are necessary to measure ecosystem service supply over the landscape include above‐ and belowground living biomass, litter and dead biomass, plant cover, soil nutrient, and soil water content (Table 2 and Figure S1). In order to simulate these stocks, nutrient, hydrological, and vegetation processes are calculated for each grid cell and/or soil layer driven by plant and soil traits and other internal (i.e., the outcome of other processes) as well as external drivers (e.g., weather conditions) (see Figure S1). We briefly describe these inter‐related processes below and provide references for readers who wish to know further details.

**Table 2:** Overview of the desired ecosystem services in the case study and how they will be measured from the simulated ecosystem and which model stocks will be considered to allow their quantification

Ecosystem service	Ecosystem measure	Model stocks
Carbon sequestration	Sum of sequestered carbon in biomass and soil	Aboveground living biomass
		Belowground living biomass
		Litter/dead biomass
		Soil carbon content
Nutrient supply	Sum of available nutrients for plants	Soil nutrient content
Erosion control	Total root fraction in the upper layer	Belowground living biomass in the upper layer
	Total vegetation cover	Plant cover
Invasion resistance	Invasive plant cover (in relation to total vegetation cover)	Invasive plant individuals
		Plant cover
Fire control	Plant functional diversity of fire strategy traits	Plant individuals with fire traits (e.g., resprouter vs. reseeder, flammability)
		Plant cover
Water retention	Total soil water content	Soil water content

#### 3.1.2 Vegetation processes

Vegetation processes capture the entire life cycle of individual woody plants distributed over the landscape and include processes such as germination/establishment, growth, reproduction/dispersal, mortality, and where applicable recovery after fire (see further description in e.g., Smith, Prentice, & Sykes, 2001). As we account for space, overlapping among neighboring individuals (above‐ as well as belowground) is explicitly modeled and thereby competition or facilitation for water, nutrients, and light is considered. All processes depend on plant‐specific traits (e.g., leaf longevity, rooting depth) and are driven by soil moisture (as a result of hydrological processes), nutrients (as a result of nutrient processes), and actual weather conditions (either measured time series or time series generated from climate data). In addition to woody plants, the herbaceous understorey could also be modeled (Landuyt et al., 2018), as this may determine, for example, recruitment success of the woody plants, as well as being important for fire dynamics.

#### 3.1.3 Hydrological processes

We simulate soil moisture dynamics by calculating all relevant hydrological processes (e.g., infiltration, runoff, drainage, evapotranspiration) for the different soil layers in the grid cells (see further description in e.g., Tietjen, Zehe, & Jeltsch, 2009). These processes depend on soil properties and topography, weather, and plant properties (from vegetation processes).

#### 3.1.4 Nutrient cycling processes

Nutrient cycling processes (e.g., decomposition, denitrification, nitrification) and nutrient fluxes between the plant and soil compartment (e.g., nitrogen uptake, soil nutrient input, leaching) are calculated for each grid cell dependent on soil properties, soil moisture, plant properties (as a result of vegetation processes) (see further information on this relationship in e.g., Everard, Seabloom, Harpole, & de Mazancourt, 2009), actual temperature conditions, and nitrogen deposition (time series data on nitrogen deposition) (see further description in e.g., Wu, McGechan, McRoberts, Baddeley, & Watson, 2007). We are focusing on only nitrogen processes as Mediterranean‐type ecosystems are primarily nitrogen‐limited. However, if necessary, the model could also be extended by considering other nutrients such as phosphorus (e.g., Daroub, Gerakis, Ritchie, Friesen, & Ryan, 2003).

A challenge during this step is that processes can act on different temporal or spatial scales (e.g., water processes act on much smaller scales than vegetation processes). However, this challenge can be approached using a modular setting (such as used in Johnson et al., 2008 or Tietjen et al., 2010), which calculates processes in separate submodels running on different temporal and spatial resolutions. During this step, we have additional measurements of plant traits not already characterized, as well as measuring soil moisture dynamics in different soil layers, to allow for a thorough model parameterization and validation. Necessary parameters that cannot be measured due to restricted resources (e.g., specific rooting depth of plant species) will be gathered from data bases (e.g., TRY: Kattge et al., 2011) or parameterized through calibration, such that model outputs match measured stocks and processes (pattern‐oriented modeling: e.g., Grimm et al., 2005; Bayesian methods: e.g., Hartig et al., 2012).

### 3.2 Step 2: Model validation and testing

For model validation, the model is parameterized and initialized based on the settings of the treatments in the Ridgefield experiment, which includes the actual spatial distribution of the individual plants, their traits and initial structure (e.g., above‐ and belowground biomass), the soil texture, and topography of the treatment plots across the site. The model should then be run under the same weather and nitrogen deposition time series as in the field experiments. Simulated soil moisture dynamics are compared to measured dynamics of the Ridgefield experiment (see Figure 3, Step 2). If there is a low root‐mean‐square deviation (also called RMSD) between measured and simulated soil moisture data, all model processes determining soil moisture can be seen as validated at least with respect to the outcome of the soil moisture. All main stocks that are used for quantifying the ecosystem services (Table 2) should be validated whether the processes have not been validated already elsewhere. As such, the simulated biomass of all species, the amount of soil carbon, and soil nitrogen could be compared to actual data.

### 3.3 Step 3: Simulation experiments of scenarios and restoration options

In the following, we demonstrate how the simulation experiments can be constructed and evaluated to answer our research questions (Figures 2 and 3, Step 3).

#### 3.3.1 Which relationships among ecosystem services result from reasonable plant trait compositions under current environmental conditions?

For the Ridgefield experiment, eight woody plant species (*Eucalyptus loxophleba* ssp*. loxophleba, E. astringens*,* Acacia acuminata*,* A. microbotrya*,* Banksia sessilis*,* Hakea lissocarpha*,* Calothamnus quadrifidus*, and *Callistemon phoeniceus*) with different traits were planted in a complete randomized block design (in each block: similar soil type, aspect, and soil moisture) of ten plant assemblages. Plant species were selected based on their nutrient acquisition strategy, growth form and size, rooting depth, flower color, and bloom time. Plant assemblages were chosen to represent increasing functional and species richness. For all treatments, ecosystem services such as carbon sequestration, biotic resistance toward invading species, nutrient cycling, biodiversity maintenance, and pollination are regularly evaluated via different absolute as well as proxy measurements (detailed description of the field experiment in Perring et al., 2012).

We complement the field experiment by simulating a full factorial design, in which more than eight plant species or plant functional types are integrated, starting from their seedling stage. In the simulation experiment, the same ecosystem services are quantified by ecosystem measures similar to those used in the field experiment (Table 2). Additional plant species compositions are simulated by artificially assembling reasonable trait compositions that include often found covariations (e.g., trade‐offs between seed size vs. seed number) in repeated long‐term simulations covering at least two life cycles of the target species and accounting for random processes such as weather events and plant dispersal. Also, to assess the effect of trait variation and covariation on selected functions/services, either single trait changes (via local sensitivity analyses) or joint trait changes (via global sensitivity analyses) could be tested (see general Step 2).

We assess the supply of multiple ecosystem service supply for current environmental conditions. We evaluate trade‐offs or synergies between the provision of selected ecosystem services by pairwise comparisons. As well as pairwise comparisons, the multifunctionality of the system could be assessed with various methodologies, for example, threshold approaches (Byrnes et al., 2014).

#### 3.3.2 What are the indirect and direct impacts of changing environmental factors on ecosystem functioning? And which simultaneous effects of multiple changing environmental factors on ecosystem functioning and service provisioning are nonadditive and why?

To assess the indirect and the direct effects of changing environmental factors (such as nitrogen deposition, climate), the separate impact of a realistic change in each environmental factor is assessed for various species assemblages. For each environmental change, two scenarios are calculated: (1) to include only indirect effects, all direct environmental effects are kept on a constant level (e.g., the direct effect of temperature on the growth function), while community change occurs through altered leaf and/or root traits as the simulation progresses, and (2) to assess the additional impact of direct effects, the same simulations are run accounting for both direct and indirect effects.

To assess whether the effects of changing environmental factors are additive or not, all changing environmental factors should be run separately and in different combinations in a full factorial design. Scenario outcomes of multiple changing factors are compared with the cumulative outcomes of the individual factors. For all analyses, the provision of ecosystem services is evaluated as described in question 1, that is, either via pairwise comparisons or indices of multifunctionality.

#### 3.3.3 Are there plant trait compositions that provide a resilient supply of multiple ecosystem services under global change?

For the Ridgefield experiment, the ten plant assemblages are treated with or without nitrogen deposition and invasive plant species (via herbaceous biomass removal) in a nested split‐plot design. Simulation experiments accounting for more than these two changing factors (i.e., also changes in climatic conditions) complement the field experiment. In particular, we run long‐term simulations for projected changes of multiple environmental factors. Plant trait compositions are detected that optimize the current and future supply of multiple ecosystem services. Additionally, we assess whether service delivery over time is resilient (i.e., is maintained either through resisting change or recovering from change back to desired levels).

Our model approach explicitly accounts for site‐specific characteristics of the Ridgefield experiment such as soil type, topography, and land use legacy. Through the use of a case study such as this, we can suggest site‐specific species assemblages that restore multiple ecosystem services and improve their resilient supply for degraded Mediterranean‐type ecosystems in South West and South Australia with similar characteristics. Modifying site conditions, for example, soil type, while keeping other environmental factors constant, would allow us to investigate whether recommendations change for such different conditions. In addition, we will improve our theoretical understanding of the multilayered relationship consisting of multiple environmental factors influencing multiple plant traits and ecosystem functions/services. In a follow‐up analysis, we can advance the knowledge about Mediterranean‐type ecosystems in general, for example, by testing whether trade‐offs among ecosystem services are site‐specific and related to particular plant trait attribute values, or transferable to the entire Mediterranean biome. To this end, model experiments (Step 3) can be rerun for different Mediterranean sites around the world after the model has been retested and validated for the respective sites (Step 2). In addition, a systematic comparison between Mediterranean‐type ecosystems can be conducted that evaluates (1) if similar trait values lead to a maximization of specific ecosystem services, and (2) if the trade‐offs between services are similar for different regions with different characteristics and species pools. Future work could also consider whether there are global change factors, for example, chemical pollutants, ecosystem functions, and/or services that deserve greater attention when planning and assessing restoration.

## 4 CONCLUSION

To our knowledge, there are no mechanistic trait‐based approaches that investigate relationships among multiple ecosystem services under the simultaneous impact of more than two changing environmental factors. We believe that our proposed integrative framework will close the gaps and thereby further the research field of restoration ecology to ultimately improve outcomes of the global restoration agenda. Our framework can contribute to trait‐based research with respect to theory development and testing. Most importantly, our framework could for a given site suggest plant species compositions that could maximize the supply of multiple ecosystem services in the long term for given environmental changes. Through this endeavor, it could directly assist restoration efforts toward resilient multifunctional ecosystems. Alternatively, by not only simulating a single ecosystem but instead multiple connected ecosystems representing a landscape, it can highlight when integrating multiple restored ecosystems better provides desired, resilient, multifunctional landscapes as opposed to one single multifunctional ecosystem “type”. Reaching the restoration goal of resilient supply of multiple ecosystem services in a changing environment needs integration of different research approaches. Our proposed framework provides a critical link between simulation modeling and in the ground research, to ultimately allow scientists, policy makers, and stakeholders to deliver the required improved restoration outcomes globally.

## CONFLICT OF INTEREST

None declared.

## AUTHOR CONTRIBUTIONS

SF conducted the literature review, prepared all figures and tables, and wrote the first draft of the manuscript. All authors contributed to the conception and design of the study of the figures and tables, contributed critically to the drafts, and gave final approval for publication.

## References

[ece34043-bib-0001] Ashbacher, A. C. , & Cleland, E. E. (2015). Native and exotic plant species show differential growth but similar functional trait responses to experimental rainfall. Ecosphere, 6(11), art245 https://doi.org/10.1890/ES15-00059.1

[ece34043-bib-0002] Bardgett, R. D. , Mommer, L. , & de Vries, F. T. (2014). Going underground: Root traits as drivers of ecosystem processes. Trends in Ecology & Evolution, 29(12), 692–699. https://doi.org/10.1016/j.tree.2014.10.006 25459399 10.1016/j.tree.2014.10.006

[ece34043-bib-0003] De Bello, F. , Lavorel, S. , Díaz, S. , Harrington, R. , Cornelissen, J. H. C. , Bardgett, R. D. , … Harrison, P. A. (2010). Towards an assessment of multiple ecosystem processes and services via functional traits. Biodiversity and Conservation, 19, 2873–2893. https://doi.org/10.1007/s10531-010-9850-9

[ece34043-bib-0004] Bennett, E. M. , Peterson, G. D. , & Gordon, L. J. (2009). Understanding relationships among multiple ecosystem services. Ecology Letters, 12, 1394–1404. https://doi.org/10.1111/j.1461-0248.2009.01387.x 19845725 10.1111/j.1461-0248.2009.01387.x

[ece34043-bib-0005] Bugalho, M. N. , Caldeira, M. C. , Pereira, J. S. , Aronson, J. , & Pausas, J. G. (2011). Mediterranean cork oak savannas require human use to sustain biodiversity and ecosystem services. Frontiers in Ecology and the Environment, 9(5), 278–286. https://doi.org/10.1890/100084

[ece34043-bib-0006] Bullock, J. M. , Aronson, J. , Newton, A. C. , Pywell, R. F. , & Rey‐Benayas, J. M. (2011). Restoration of ecosystem services and biodiversity: Conflicts and opportunities. Trends in Ecology & Evolution, 26(10), 541–549. https://doi.org/10.1016/j.tree.2011.06.011 21782273 10.1016/j.tree.2011.06.011

[ece34043-bib-0007] Byrnes, J. E. K. , Gamfeldt, L. , Isbell, F. , Lefcheck, J. S. , Griffin, J. N. , Hector, A. , & Emmett Duffy, J. (2014). Investigating the relationship between biodiversity and ecosystem multifunctionality: Challenges and solutions. Methods in Ecology and Evolution, 5, 111–124. https://doi.org/10.1111/2041-571210X.12143

[ece34043-bib-0008] Cochrane, A. , Hoyle, G. L. , Yates, C. J. , Wood, J. , & Nicotra, A. B. (2015). The phenotypic response of co‐occurring Banksia species to warming and drying. Plant Ecology, 216, 27–39. https://doi.org/10.1007/s11258-014-0414-z

[ece34043-bib-0009] Cowling, R. M. , Rundel, P. W. , Lamont, B. B. , Arroyo, M. K. , & Arianoutsou, M. (1996). Plant diversity in mediterranean‐climate regions. TREE, 11, 362–366. 21237880 10.1016/0169-5347(96)10044-6

[ece34043-bib-0010] Daily, G. C. (1997). Nature's services: Societal dependence on natural ecosystems. Washington, DC: Island Press.

[ece34043-bib-0011] Daroub, S. H. , Gerakis, A. , Ritchie, J. T. , Friesen, D. K. , & Ryan, J. (2003). Development of a soil‐plant phosphorus simulation model for calcareous and weathered tropical soils. Agricultural Systems, 76(3), 1157–1181. https://doi.org/10.1016/S0308-521X(02)00082-3

[ece34043-bib-0012] Esther, A. , Groeneveld, J. , Enright, N. J. , Miller, B. P. , Lamont, B. B. , Perry, G. L. W. , … Jeltsch, F. (2011). Low‐dimensional trade‐offs fail to explain richness and structure in species‐rich plant communities. Theoretical Ecology, 4, 495–511. https://doi.org/10.1007/s12080-010-0092-y

[ece34043-bib-0013] Everard, K. , Seabloom, E. W. , Harpole, W. S. , & de Mazancourt, C. (2009). Plant water use affects competition for nitrogen: Why drought favors invasive species in California. The American Naturalist, 175(1), 85–97. https://doi.org/10.1086/648557 10.1086/648557 19916786

[ece34043-bib-0014] Funk, J. L. , Cleland, E. E. , Suding, K. N. , & Zavaleta, E. S. (2008). Restoration through reassembly: Plant traits and invasion resistance. Trends in Ecology & Evolution, 23(12), 695–703. https://doi.org/10.1016/j.tree.2008.07.013 18951652 10.1016/j.tree.2008.07.013

[ece34043-bib-0015] Funk, J. L. , Larson, J. E. , Ames, G. M. , Butterfield, B. J. , Cavender‐Bares, J. , Firn, J. , … Wright, J. (2017). Revisiting the Holy Grail: Using plantfunctional traits to understand ecological processes. Biological Reviews, 92, 1156–1173. https://doi.org/10.1111/brv.12275 27103505 10.1111/brv.12275

[ece34043-bib-0016] Fyllas, N. M. , & Troumbis, A. Y. (2009). Simulating vegetation shifts in north‐eastern Mediterranean mountain forests under climatic change scenarios. Global Ecology and Biogeography, 18, 64–77. https://doi.org/10.1111/j.1466-8238.2008.00419.x

[ece34043-bib-0017] Garnier, E. , Bellmann, A. , Navas, M.‐L. , Roumet, C. , & Laurent, G. (2004). The leaf‐height‐seed plant ecology strategy scheme as applied to species from a Mediterranean old‐field succession In Arianoutsou M. & Papanastasis V. P. (Eds.), Proceedings of the 10th MEDECOS Conference, Rhodes Island, Greece (p. 16). Rotterdam, NL.

[ece34043-bib-0018] Garnier, E. , Navas, M.‐L. , & Grigulis, K. (2016). Plant functional diversity: Organism traits, community structure and ecosystem properties. Oxford, UK: Oxford University Press.

[ece34043-bib-0019] Godoy, O. , Castro‐Díez, P. , Van Logtestijn, R. S. P. , Cornelissen, J. H. C. , & Valladares, F. (2010). Leaf litter traits of invasive species slow down decomposition compared to Spanish natives: A broad phylogenetic comparison. Oecologia, 162, 781–790. https://doi.org/10.1007/s00442-009-1512-9 20155374 10.1007/s00442-009-1512-9

[ece34043-bib-0020] Gratani, L. , Varone, L. , Ricotta, C. , & Catoni, R. (2013). Mediterranean shrublands carbon sequestration: Environmental and economic benefits. Mitigation and Adaptation Strategies for Global Change, 18, 1167–1182. https://doi.org/10.1007/s11027-012-9415-1

[ece34043-bib-0021] Grimm, V. , Revilla, E. , Berger, U. , Jeltsch, F. , Mooij, W. M. , Railsback, S. F. , … DeAngelis, D. L. (2005). Pattern‐oriented modeling of agent‐based complex systems: Lessons from ecology. Science, 310, 987–991. https://doi.org/10.1126/science.1116681 16284171 10.1126/science.1116681

[ece34043-bib-0022] Hartig, F. , Dyke, J. , Hickler, T. , Higgins, S. I. , O'Hara, R. B. , Scheiter, S. , & Huth, A. (2012). Connecting dynamic vegetation models to data – an inverse perspective. Journal of Biogeography, 39, 2240–2252. https://doi.org/10.1111/j.1365-2699.2012.02745.x

[ece34043-bib-0023] Hobbs, R. J. (1998). Impacts of land use on biodiversity in Southwestern Australia In Rundel P. W., Montenegro G., & Jaksic F. (Eds.), Landscape disturbance and biodiversity in Mediterranean‐type ecosystems (pp. 81–106). Berlin, Germany: Springer.

[ece34043-bib-0024] Holling, C. S. (1973). Resilience and stability of ecological systems. Annual Review of Ecology and Systematics, 4, 1–23. https://doi.org/10.1146/annurev.es.04.110173.000245

[ece34043-bib-0025] Hopfe, C. J. , & Hensen, J. L. M. (2011). Uncertainty analysis in building performance simulation for design support. Energy and Buildings, 43, 2798–2805. https://doi.org/10.1016/j.enbuild.2011.06.034

[ece34043-bib-0026] IPCC (2013). Climate Change 2013: The physical science basis. Contribution of Working Group I to the Fifth Assessment Report of the Intergovernmental Panel on Climate Change. In T.F. Stocker, D. Qin, G.‐K. Plattner, M. Tignor, S.K. Allen, J. Boschung, A. Nauels, Y. Xia, V. Bex, & P.M. Midgley (Eds.). Cambridge, United Kingdom and New York, NY: Cambridge University Press.

[ece34043-bib-0027] Johnson, I. R. , Chapman, D. F. , Snow, V. O. , Eckard, R. J. , Parsons, A. J. , Lambert, M. G. , & Cullen, B. R. (2008). DairyMod and EcoMod: Biophysical pasture‐simulation models for Australia and New Zealand. Australian Journal of Experimental Agriculture, 48, 621–631. https://doi.org/10.1071/EA07133

[ece34043-bib-0028] Kattge, J. , Díaz, S. , Lavorel, S. , Prentice, I. C. , Leadley, P. , Bönisch, G. , … Wirth, C. (2011). TRY – a global database of plant traits. Global Change Biology, 17, 2905–2935. https://doi.org/10.1111/j.1365-2486.2011.02451.x

[ece34043-bib-0029] Landuyt, D. , Perring, M. P. , Seidl, R. , Taubert, F. , Verbeeck, H. , & Verheyen, K. (2018). Modelling understorey dynamics in temperate forests under global change–Challenges and perspectives. Perspectives in Plant Ecology, Evolution and Systematics, 31, 44–54. https://doi.org/10.1016/j.ppees.2018.01.002 10.1016/j.ppees.2018.01.002 PMC5884426 29628800

[ece34043-bib-0030] Laughlin, D. C. (2014a). Applying trait‐based models to achieve functional targets for theory‐driven ecological restoration. Ecology Letters, 17, 771–784. https://doi.org/10.1111/ele.12288 24766299 10.1111/ele.12288

[ece34043-bib-0031] Laughlin, D. C. (2014b). The intrinsic dimensionality of plant traits and its relevance to community assembly. Journal of Ecology, 102, 186–193. https://doi.org/10.1111/1365-2745.12187

[ece34043-bib-0032] Lavergne, S. , Garnier, E. , & Debussche, M. (2003). Do rock endemic and widespread plant species differ under the Leaf‐Height‐Seed plant ecology strategy scheme? Ecology Letters, 6, 398–404. https://doi.org/10.1046/j.1461-0248.2003.00456.x

[ece34043-bib-0033] Lavorel, S. , & Garnier, E. (2002). Predicting changes in community composition and ecosystem functioning from plant traits: Revisiting the Holy Grail. Functional Ecology, 16, 545–556. https://doi.org/10.1046/j.1365-2435.2002.00664.x

[ece34043-bib-0034] LeRoy, C. J. , Wymore, A. S. , Davis, R. , & Marks, J. C. (2014). Indirect influences of a major drought on leaf litter quality and decomposition in a southwestern stream. Fundamental and Applied Limnology, 184(1), 1–10. https://doi.org/10.1127/1863-9135/2014/0505

[ece34043-bib-0035] Mace, G. M. , Norris, K. , & Fitter, A. H. (2012). Biodiversity and ecosystem services: A multilayered relationship. Trends in Ecology & Evolution, 27(1), 19–26. https://doi.org/10.1016/j.tree.2011.08.006 21943703 10.1016/j.tree.2011.08.006

[ece34043-bib-0036] McGill, B. , Enquist, B. , Weiher, E. , & Westoby, M. (2006). Rebuilding community ecology from functional traits. Trends in Ecology & Evolution, 21(4), 178–185. https://doi.org/10.1016/j.tree.2006.02.002 16701083 10.1016/j.tree.2006.02.002

[ece34043-bib-0037] MEA (Millennium Ecosystem Assessment) (2005). Ecosystems and human well‐being: Synthesis. Washington, DC: Island Press.

[ece34043-bib-0038] Médail, F. , & Quézel, P. (1997). Hot‐spots analysis for conservation of plant biodiversity in the Mediterranean basin. Annals of the Missouri Botanical Garden, 84(1), 112–127.

[ece34043-bib-0039] Medrano, H. , Flexas, J. , & Galmés, J. (2009). Variability in water use efficiency at the leaf level among Mediterranean plants with different growth forms. Plant and Soil, 317, 17–29. https://doi.org/10.1007/s11104-008-9785-z

[ece34043-bib-0040] Meyer‐Grünefeldt, M. , Friedrich, U. , Klotz, M. , Von Oheimb, G. , & Härdtle, W. (2015). Nitrogen deposition and drought events have non‐additive effects on plant growth – Evidence from greenhouse experiments. Plant Biosystems, 149, 424–432. https://doi.org/10.1080/11263504.2013.853699

[ece34043-bib-0041] Moore, A. D. , & Noble, I. R. (1990). An individualistic model of vegetation stand dynamics. Journal of Environmental Management, 31, 61–81.

[ece34043-bib-0042] Mouillot, F. , Rambal, S. , & Lavorel, S. (2001). A generic process‐based SImulator for meditERRanean landscApes (SIERRA): Design and validation exercises. Forest Ecology and Management, 147, 75–97. https://doi.org/10.1016/S0378-1127(00)00432-1

[ece34043-bib-0043] Myers, N. (1990). The biodiversity challenge: Expanded hot‐spots analysis. The Environmentalist, 10, 243–256. https://doi.org/10.1007/BF02239720 12322583 10.1007/BF02239720

[ece34043-bib-0044] Oreskes, N. , Shrader‐Frechette, K. , & Belitz, K. (1994). Verification, validation, and confirmation of numerical models in the earth sciences. Science, 263, 641–646. https://doi.org/10.1126/science.263.5147.641 17747657 10.1126/science.263.5147.641

[ece34043-bib-0045] Pausas, J. G. (1999). Response of plant functional types to changes in the fire regime in Mediterranean ecosystems: A simulation approach. Journal of Vegetation Science, 10, 717–722.

[ece34043-bib-0046] Pérez‐Camacho, L. , Rebollo, S. , Hernández‐Santana, V. , García‐Salgado, G. , Pavón‐García, J. , & Gómez‐Sal, A. (2012). Plant functional trait responses to interannual rainfall variability, summer drought and seasonal grazing in Mediterranean herbaceous communities. Functional Ecology, 26, 740–749. https://doi.org/10.1111/j.1365-2435.2012.01967.x

[ece34043-bib-0047] Perring, M. P. , Standish, R. J. , Hulvey, K. B. , Lach, L. , Morald, T. K. , Parsons, R. , … Hobbs, R. J. (2012). The Ridgefield multiple ecosystem services experiment: Can restoration of former agricultural land achieve multiple outcomes? Agriculture, Ecosystems & Environment, 163, 14–27. https://doi.org/10.1016/j.agee.2012.02.016

[ece34043-bib-0048] Perring, M. P. , Standish, R. J. , Price, J. N. , Craig, M. D. , Erickson, T. E. , Ruthrof, K. X. , … Hobbs, R. J. (2015). Advances in restoration ecology: Rising to the challenges of the coming decades. Ecosphere, 6(8), art131 https://doi.org/10.1890/ES15-00121.1

[ece34043-bib-0049] Pierce, L. L. , Running, S. W. , & Walker, J. (1994). Regional‐scale relationships of leaf area index to specific leaf area and leaf nitrogen content. Ecological Applications, 4(2), 313–321.

[ece34043-bib-0050] Prieto, I. , Roumet, C. , Cardinael, R. , Dupraz, C. , Jourdan, C. , Kim, J. H. , … Stokes, A. (2015). Root functional parameters along a land‐use gradient: Evidence of a community‐level economics spectrum. Journal of Ecology, 103, 361–373. https://doi.org/10.1111/1365-2745.12351

[ece34043-bib-0051] Reuter, H. , Jopp, F. , Breckling, B. , Lange, C. , & Weigmann, G. (2011). How valid are model results? Assumptions, validity range and documentation In Jopp F., Reuter H., & Breckling B. (Eds.), Modelling complex ecological dynamics – An introduction into ecological modelling (pp. 323–340). Berlin, Heidelberg, Germany: Springer.

[ece34043-bib-0052] Rey Benayas, J. M. , Newton, A. C. , Diaz, A. , & Bullock, J. M. (2009). Enhancement of biodiversity and ecosystem services by ecological restoration: A meta‐analysis. Science, 325, 1121–1124. https://doi.org/10.1007/s10021-012-9552-0 19644076 10.1126/science.1172460

[ece34043-bib-0053] Ruget, F. , Brisson, N. , Delécolle, R. , & Faivre, R. (2002). Sensitivity analysis of a crop simulation model, STICS, in order to choose the main parameters to be estimated. Agronomie, 22, 133–158. https://doi.org/10.1051/agro:2002009

[ece34043-bib-0054] Ruiz‐Benito, P. , Gómez‐Aparicio, L. , Paquette, A. , Messier, C. , Kattge, J. , & Zavala, M. A. (2014). Diversity increases carbon storage and tree productivity in Spanish forests. Global Ecology and Biogeography, 23, 311–322. https://doi.org/10.1111/geb.12126

[ece34043-bib-0055] Rustad, L. , Campbell, J. , Marion, G. , Norby, R. , Mitchell, M. , Hartley, A. , … Gurevitch, J. (2001). A meta‐analysis of the response of soil respiration, net nitrogen mineralization, and aboveground plant growth to experimental ecosystem warming. Oecologia, 126(4), 543–562. https://doi.org/10.1007/s004420000544 28547240 10.1007/s004420000544

[ece34043-bib-0056] Sala, O. E. (2000). Global biodiversity scenarios for the year 2100. Science, 287, 1770–1774. https://doi.org/10.1126/science.287.5459.1770 10710299 10.1126/science.287.5459.1770

[ece34043-bib-0057] Sargent, R. G. (2013). Verification and validation of simulation models. Journal of Simulation, 7, 12–24. https://doi.org/10.1057/jos.2012.20

[ece34043-bib-0058] Sariyildiz, T. , Anderson, J. M. , & Kucuk, M. (2005). Effects of tree species and topography on soil chemistry, litter quality, and decomposition in Northeast Turkey. Soil Biology and Biochemistry, 9, 1695–1706. https://doi.org/10.1016/j.soilbio.2005.02.004

[ece34043-bib-0059] SER (Society for Ecological Restoration) (2004). The SER International Primer on Ecological Restoration. In Society for Ecological Restoration International Science & Policy Working Group (Eds.). Society for Ecological Restoration International. Retrieved from http://www.ser.org & Tucson.

[ece34043-bib-0060] Shackelford, N. , Hobbs, R. J. , Burgar, J. M. , Erickson, T. E. , Fontaine, J. B. , Laliberté, E. , … Standish, R. J. (2013). Primed for change: Developing ecological restoration for the 21st century. Restoration Ecology, 21(3), 297–304. https://doi.org/10.1111/rec.12012

[ece34043-bib-0061] Simpson, K. J. , Ripley, B. S. , Christin, P.‐A. , Belcher, C. M. , Lehmann, C. E. R. , Thomas, G. H. , & Osborne, C. P. (2016). Determinants of flammability in savanna grass species. Journal of Ecology, 104, 138–148. https://doi.org/10.1111/1365-2745.12503 26877549 10.1111/1365-2745.12503 PMC4738432

[ece34043-bib-0062] Smith, B. , Prentice, I. C. , & Sykes, M. T. (2001). Representation of vegetation dynamics in modelling of terrestrial ecosystems: Comparing two contrasting approaches within European climate space. Global Ecology and Biogeography, 10, 621–637. https://doi.org/10.1046/j.1466-822X.2001.t01-1-00256.x

[ece34043-bib-0063] Suding, K. , Higgs, E. , Palmer, M. , Callicott, J. B. , Anderson, C. B. , Baker, M. , … Schwartz, K. Z. S. (2015). Committing to ecological restoration. Science, 348, 638–640. https://doi.org/10.1126/science.aaa4216 25953995 10.1126/science.aaa4216

[ece34043-bib-0064] Suding, K. N. , Lavorel, S. , Chapin, F. S. , Cornelissen, J. H. C. , Díaz, S. , Garnier, E. , … Navas, M.‐L. (2008). Scaling environmental change through the community‐level: A trait‐based response‐and‐effect framework for plants. Global Change Biology, 14, 1125–1140. https://doi.org/10.1111/j.1365-2486.2008.01557.x

[ece34043-bib-0065] Tietjen, B. , Jeltsch, F. , Zehe, E. , Classen, N. , Groengroeft, A. , Schiffers, K. , & Oldeland, J. (2010). Effects of climate change on the coupled dynamics of water and vegetation in drylands. Ecohydrology, 3, 226–237. https://doi.org/10.1002/eco.70

[ece34043-bib-0066] Tietjen, B. , Zehe, E. , & Jeltsch, F. (2009). Simulating plant water availability in dry lands under climate change: A generic model of two soil layers. Water Resources Research, 45, W01418 https://doi.org/10.1029/2007WR006589

[ece34043-bib-0067] Valera‐Burgos, J. , Zunzunegui, M. , & Díaz‐Barradas, M. C. (2013). Do leaf traits and nitrogen supply affect decomposability rates of three Mediterranean species growing under different competition levels? Pedobiologia, 56, 113–119. https://doi.org/10.1016/j.pedobi.2013.03.002

[ece34043-bib-0068] Vallejo, R. , Aronson, J. , Pausas, J. G. , & Cortina, J. (2001). Restoration of Mediterranean woodlands In van Andel J., & Aronson J. (Eds.), Restoration ecology: A European perspective (pp. 193–207). Oxford, UK: Blackwell Science.

[ece34043-bib-0069] Vilà‐Cabrera, A. , Martínez‐Vilalta, J. , & Retana, J. (2015). Functional trait variation along environmental gradients in temperate and Mediterranean trees. Global Ecology and Biogeography, 24, 1377–1389. https://doi.org/10.1111/geb.12379

[ece34043-bib-0070] Violle, C. , Navas, M.‐L. , Vile, D. , Kazakou, E. , Fortunel, C. , Hummel, I. , & Garnier, E. (2007). Let the concept of trait be functional!. Oikos, 116, 882–892. https://doi.org/10.1111/j.2007.0030-1299.15559.x

[ece34043-bib-0071] Westoby, M. (1998). A leaf‐height‐seed (LHS) plant ecology strategy scheme. Plant and Soil, 199, 213–227. https://doi.org/10.1023/A:1004327224729

[ece34043-bib-0072] Wu, L. , McGechan, M. B. , McRoberts, N. , Baddeley, J. A. , & Watson, C. A. (2007). SPACSYS: Integration of a 3D root architecture component to carbon, nitrogen and water cycling—Model description. Ecological Modelling, 200, 343–359. https://doi.org/10.1016/j.ecolmodel.2006.08.010

