# Supplementary material for: Integrating trait‐based empirical and modeling research to improve ecological restoration
Source: Ecol Evol. 2018 May 29;8(12):6369–80. doi: 10.1002/ece3.4043 (PMC6024147; doi:10.1002/ece3.4043)
Supplement: Supplementary file 1 [file ECE3-8-6369-s001.docx]

# Supporting Information

Assessing the state of knowledge, we performed a literature search of papers published between 2009 and 2016 in the database “Web of Science”.

First, we assessed the knowledge on the effect of plant traits on ecosystem functions and ecosystem services and trade-offs among them. To this end, we searched for publications on the key words “Mediterranean”, “plant trait”, “ecosystem function” or “ecosystem service” and/or “trade-off”. From this search examples are summarised in Table S1.

Secondly, we assessed the knowledge on effects of environmental change factors on ecosystem functioning and service provisioning. Therefore, we searched for publications on the key words “Mediterranean”, “plant trait” and on the following environmental change factors: “land use”, “invasion”, “climate warming”, “drought”, “nitrogen deposition” or “carbon dioxide”. If the resulting studies assessed the effect of one of the factors on ecosystem functions/properties and/or plant traits, the studies were sorted according to the environmental change factors studied. Affected plant traits and functions, and whether the study assessed a direct or indirect effect of environmental change were recorded and relevant papers were summarised in Table S2.

Inspection of these papers showed us that there was a gap in knowledge regarding the multi-layered relationship among environmental change, plant traits and the delivery of ecosystem services, which we briefly introduce in the paper. The main text then focuses on how this knowledge gap can be addressed through a coupled simulation model – empirical research agenda.

Table S1. Examples of trait-based approaches (since 2009) contributing to the assessment of ecosystem services in Mediterranean-type ecosystems. For several ecosystem services (italicised bold text) we have put the class of plant traits linked to specific affected functions, and the proxy measures that trait-based studies used

|  | **Plant trait** | **Affected ecosystem function** | **Affected ecosystem measure** | **References** |
| --- | --- | --- | --- | --- |
| ***Carbon sequestration*** | | | | |
|  | Leaf traits | Photosynthesis | Photosynthetic leaf surface area | Gratani et al. (2013) |
|  | Root traits | -* | Biomass, Total soil organic carbon | Butterfield and Suding (2013) |
|  | Diversity of plant traits | Carbon storage | Biomass, Species-specific carbon content | Ruiz-Benito et al. (2014) |
| ***Nutrient supply*** | | | | |
|  | Root traits | Decomposition | Decomposition rate | Birouste et al. (2012) |
| ***Erosion control*** | | | | |
|  | Stem, leaf, plant morphological traits | Soil retention | Mass of sediment trapped per unit volume | Burylo et al. (2012) |
| ***Invasion resistance*** | | | | |
|  | Diversity of plant traits | Establishment and reproduction of invaders | Number of established individuals, Number of flower heads and seeds, Total stem length | Hooper and Dukes (2010) |
| ***Fire control*** | | | | |
|  | Leaf traits | Flammability | Time to ignition, Predicted rate of fire spread, Flaming time, Combustion rate | Simpson et al. (2016) |
| ***Water retention*** | | | | |
|  | Leaf traits | Transpiration | Assimilation rate, Stomatal conductance, Transpiration rate | Medrano, Flexas, and Galmés (2009); Yates et al. (2010) |

* This study directly measured the ecosystem service and not a function.

Birouste, M., Kazakou, E., Blanchard, A., & Roumet, C. (2012). Plant traits and decomposition: are the relationships for roots comparable to those for leaves? Annals of Botany, 109, 463–472. doi: 10.1093/aob/mcr297

Burylo, M., Rey, F., Bochet, E., & Dutoit, T. (2012). Plant functional traits and species ability for sediment retention during concentrated flow erosion. Plant and Soil, 353, 135–144. doi: 10.1007/s11104-011-1017-2

Butterfield, B. J., & Suding, K. N. (2013). Single-trait functional indices outperform multi-trait indices in linking environmental gradients and ecosystem services in a complex landscape. Journal of Ecology, 101, 9–17. doi: 10.1111/1365-2745.12013

Gratani, L., Varone, L., Ricotta, C., & Catoni, R. (2013). Mediterranean shrublands carbon sequestration: environmental and economic benefits. Mitigation and Adaptation Strategies for Global Change, 18, 1167–1182. doi: 10.1007/s11027-012-9415-1

Hooper, D. U., & Dukes, J. S. (2010). Functional composition controls invasion success in a California serpentine grassland. Journal of Ecology, 98, 764–777. doi: 10.1111/j.1365-2745.2010.01673.x

Medrano, H., Flexas, J., & Galmés, J. (2009). Variability in water use efficiency at the leaf level among Mediterranean plants with different growth forms. Plant and Soil, 317, 17–29. doi: 10.1007/s11104-008-9785-z

Ruiz-Benito, P., Gómez-Aparicio, L., Paquette, A., Messier, C., Kattge, J., & Zavala, M. A. (2014). Diversity increases carbon storage and tree productivity in Spanish forests. Global Ecology and Biogeography, 23, 311–322. doi: 10.1111/geb.12126

Simpson, K. J., Ripley, B. S., Christin, P.-A., Belcher, C. M., Lehmann, C. E. R., Thomas, G. H., & Osborne, C. P. (2016). Determinants of flammability in savanna grass species. Journal of Ecology, 104, 138–148. doi: 10.1111/1365-2745.12503

Yates, M.J., Anthony Verboom, G., Rebelo, A. G., & Cramer, M. D. (2010). Ecophysiological significance of leaf size variation in Proteaceae from the Cape Floristic Region. Functional Ecology, 24, 485–492. doi: 10.1111/j.1365-2435.2009.01678.x

Table S2. Examples of trait-based approaches studying the effect of global environmental change factors (italicised bold text) on plant traits and ecosystem functions/services in Mediterranean-type ecosystems (as defined in Figure 1). Studied effects of global change are distinguished (third column) between studies that assess (i) the effect of global change on traits but not on ecosystem functioning (-), (ii) the direct effect of global change on traits and on functioning separately (without accounting for the link from plant traits to ecosystem functioning), and (iii) the effects of global change on ecosystem functions via plant traits (indirect effects). Highlighted references focus on two changing environmental factors

| **Affected plant trait** | **Studied ecosystem function/property** | **Studied effect** | **References** (see Appendix S1) |
| --- | --- | --- | --- |
| ***Land use*** | | | |
| Leaf (litter) traits | None | - | Castro et al. (2010); Targetti et al. (2013); Batriu, Ninot, and Pino (2015) |
|  | Decomposition | Indirect | Cortez et al. (2007); Castro, Fortunel, and Freitas (2010) |
|  | Decomposition, growth | Indirect | Kazakou et al. (2006) |
| Root traits | None | - | Prieto et al. (2015) |
| Other morphological traits | None | - | Lavorel, McIntyre, and Grigulis (1999); Castro et al. (2010); Pérez-Camacho et al. (2012); Gutiérrez-Girón and Gavilán (2013) |
|  | Soil moisture, compaction, nutrients | Direct | Dobarro, Pérez Carmona, and Peco (2013) |
| Reproductive traits | None | - | Lavorel, McIntyre, and Grigulis (1999); Castro et al. (2010) |
|  | Soil moisture, compaction, nutrients | Direct | Dobarro, Pérez Carmona, and Peco (2013) |
| ***Biotic invasion*** | | | |
| Leaf (litter) traits | None | - | Lloret et al. (2005); Acosta, Izzi, and Stanisci (2006); Grotkopp and Rejmánek (2007); Stanisci et al. (2010) |
|  | Decomposition | Indirect | Godoy et al. (2010) |
|  | Decomposition | Direct and indirect | LeRoy et al. (2014) |
|  | Nitrogen supply and carbon storage | Direct | Wolkovich et al. (2010) |
|  | Photosynthesis | Direct | Garcia-Serrano et al. (2009) |
|  | Growth | Direct | Domènech and Vilà (2008) |
| Root traits | None | - | Stanisci et al. (2010) |
|  | Nitrogen use, biomass | Direct | Werner et al. (2010) |
| Other morphological traits | None | - | Lloret et al. (2005); Acosta, Izzi, and Stanisci (2006) |
|  | Growth | Direct | Domènech and Vilà (2008) |
|  | Competition, nitrogen use, biomass | Direct | Werner et al. (2010) |
| Reproductive traits | None | - | Lloret et al. (2005); Acosta, Izzi, and Stanisci (2006); Godoy et al. (2009a; b); Stanisci et al. (2010); Castro-Díez et al. (2014) |
|  | Germination, emergence, establishment, growth, reproduction | Direct | Sans, Garcia-Serrano, and Afán (2004) |
| Physiological traits | Growth | Direct | Ashbacher and Cleland (2015) |
| ***Climate*** | | | |
| Leaf (litter) traits | None | - | Crescente, Gratani, and Larcher (2002); Martin-StPaul et al. (2013) |
|  | Biomass | Direct | Cochrane et al. (2015) |
|  | Growth, biomass | Indirect | Cochrane et al. (2015) |
|  | Growth | Direct | Domènech and Vilà (2008) |
|  | Competition, nitrogen use, biomass | Direct | Werner et al. (2010) |
|  | Photosynthesis | Direct | Garcia-Serrano et al. (2009) |
| Root traits | Competition, nitrogen use, biomass | Direct | Werner et al. (2010) |
| Other morphological traits | None | - | Pérez-Camacho et al. (2012); Lázaro-Nogal et al. (2013) |
|  | Growth | Direct | Domènech and Vilà (2008) |
|  | Nitrogen use, biomass | Direct | Werner et al. (2010) |
| Reproductive traits | None | - | del Cacho, Peñuelas, and Lloret (2013); Lázaro-Nogal et al. (2013); Hänel & Tielbörger (2015) |
| Physiological traits | None | - | Martin-StPaul et al. (2013); Lázaro-Nogal et al. (2013) |
|  | Growth | Direct | Ashbacher and Cleland (2015) |
| Fire strategy traits | Germination | Direct | Paula and Pausas (2008) |
| ***Nitrogen deposition*** | | | |
| Leaf (litter) traits | Organic matter and inorganic nitrogen content, nitrogen and carbon fluxes | Direct | Dias et al. (2013) |
|  | Productivity, nitrogen and carbon fluxes | Direct | Vourlitis, Pasquini, and Mustard (2009) |
|  | Decomposition | Indirect | Kazakou et al. (2009); Valera-Burgos, Zunzunegui, and Díaz-Barradas (2013) |
| ***Atmospheric CO_2_*** | | | |
| Leaf (litter) traits | Decomposition | Direct and indirect | Gahrooee (1998) |
| Reproductive traits | Emergence success, biomass | Direct | Grünzweig and Dumbur (2012) |

Acosta, A., Izzi, C. F., & Stanisci, A. (2006). Comparison of native and alien plant traits in Mediterranean coastal dunes. Community Ecology, 7(1), 35–41. doi: 10.1556/ComEc.7.2006.1.4

Ashbacher, A. C., & Cleland, E. E. (2015). Native and exotic plant species show differential growth but similar functional trait responses to experimental rainfall. Ecosphere, 6(11), art245. doi: 10.1890/ES15-00059.1

Batriu, E., Ninot, J. M., & Pino, J. (2015). Filtering of plant functional traits is determined by environmental gradients and by past land use in a Mediterranean coastal marsh. Journal of Vegetation Science, 26, 492–500. doi: 10.1111/jvs.12251

del Cacho, M., Peñuelas, J., & Lloret, F. (2013). Reproductive output in Mediterranean shrubs under climate change experimentally induced by drought and warming. Perspectives in Plant Ecology, Evolution and Systematics, 15, 319–327. doi: 10.1016/j.ppees.2013.07.001

Castro-Díez, P., Valle, G., González-Muñoz, N., & Alonso, Á. (2014). Can the Life-History Strategy Explain the Success of the Exotic Trees Ailanthus altissima and Robinia pseudoacacia in Iberian Floodplain Forests? PLoS ONE, 9(6), e100254. doi: 10.1371/journal.pone.0100254

Castro, H., Fortunel, C., & Freitas, H. (2010). Effects of land abandonment on plant litter decomposition in a Montado system: relation to litter chemistry and community functional parameters. Plant and Soil, 333, 181–190. doi: 10.1007/s11104-010-0333-2

Castro, H., Lehsten, V., Lavorel, S., & Freitas, H. (2010). Functional response traits in relation to land use change in the Montado. Agriculture, Ecosystems & Environment, 137, 183–191. doi: 10.1016/j.agee.2010.02.002

Cochrane, A., Hoyle, G. L., Yates, C. J., Wood, J., & Nicotra, A. B. (2015). The phenotypic response of co-occurring Banksia species to warming and drying. Plant Ecology, 216, 27–39. doi: 10.1007/s11258-014-0414-z

Cortez, J., Garnier, E., Pérez-Harguindeguy, N., Debussche, M., & Gillon, D. (2007). Plant traits, litter quality and decomposition in a Mediterranean old-field succession. Plant and Soil, 296, 19–34. doi: 10.1007/s11104-007-9285-6

Crescente, M. F., Gratani, L., & Larcher, W. (2002). Shoot growth efficiency and production of Quercus ilex L. in different climates. Flora - Morphology, Distribution, Functional Ecology of Plants, 197, 2–9. doi: 10.1078/0367-2530-00007

Dias, T., Oakley, S., Alarcón-Gutiérrez, E., Ziarelli, F., Trindade, H., Martins-Loução, M. A., … Cruz, C. (2013). N-driven changes in a plant community affect leaf-litter traits and may delay organic matter decomposition in a Mediterranean maquis. Soil Biology and Biochemistry, 58, 163–171. doi: 10.1016/j.soilbio.2012.10.027

Dobarro, I., Pérez Carmona, C., & Peco, B. (2013). Dissecting the Effects of Simulated Cattle Activity on Floristic Composition and Functional Traits in Mediterranean Grasslands. PLoS ONE, 8(11), e79822. doi: 10.1371/journal.pone.0079822 Editor:

Domènech, R., & Vilà, M. (2008). Response of the invader Cortaderia selloana and two coexisting natives to competition and water stress. Biological Invasions, 10, 903–912. doi: 10.1007/s10530-008-9243-0

Gahrooee, F. R. (1998). Impacts of elevated atmospheric CO2 on litter quality, litter decomposability and nitrogen turnover rate of two oak species in a Mediterranean forest ecosystem. Global Change Biology, 4, 667–677. doi: 10.1046/j.1365-2486.1998.00187.x

Garcia-Serrano, H., Caño, L., Escarré, J., Fleck, I., & Sans, F.X. (2009). Physiological comparison of alien Senecio inaequidens and S. pterophorus and native S. malacitanus: Implications for invasion. Flora - Morphology, Distribution, Functional Ecology of Plants, 204, 445–455. doi: 10.1016/j.flora.2008.05.006

Godoy, O., Castro-Díez, P., Van Logtestijn, R. S. P., Cornelissen, J. H. C., & Valladares, F. (2010). Leaf litter traits of invasive species slow down decomposition compared to Spanish natives: a broad phylogenetic comparison. Oecologia, 162, 781–790. doi: 10.1007/s00442-009-1512-9

Godoy, O., Castro-Díez, P., Valladares, F., & Costa-Tenorio, M. (2009a). Different flowering phenology of alien invasive species in Spain: evidence for the use of an empty temporal niche? Plant Biology, 11, 803–811. doi: 10.1111/j.1438-8677.2008.00185.x

Godoy, O., Richardson, D. M., Valladares, F., & Castro-Diez, P. (2009b). Flowering phenology of invasive alien plant species compared with native species in three Mediterranean-type ecosystems. Annals of Botany, 103, 485–494. doi: 10.1093/aob/mcn232

Grotkopp, E., & Rejmánek, M. (2007). High seedling relative growth rate and specific leaf area are traits of invasive species: phylogenetically independent contrasts of woody angiosperms. American Journal of Botany, 94(4), 526–532. doi: 10.3732/ajb.94.4.526

Grünzweig, J. M., & Dumbur, R. (2012). Seed traits, seed-reserve utilization and offspring performance across pre-industrial to future CO2 concentrations in a Mediterranean community. Oikos, 121, 579–588. doi: 10.1111/j.1600-0706.2011.19770.x

Gutiérrez-Girón, A., & Gavilán, R. (2013). Plant functional strategies and environmental constraints in Mediterranean high mountain grasslands in central Spain. Plant Ecology & Diversity, 6(3-4), 435–446. doi: 10.1080/17550874.2013.783641

Hänel, S., & Tielbörger, K. (2015). Phenotypic response of plants to simulated climate change in a long-term rain-manipulation experiment: a multi-species study. Oecologia, 177, 1015–1024. doi: 10.1007/s00442-015-3231-8

Kazakou, E., Vile, D., Shipley, B., Gallet, C., & Garnier, E. (2006). Co-variations in litter decomposition, leaf traits and plant growth in species from a Mediterranean old-field succession. Functional Ecology, 20, 21–30. doi: 10.1111/j.1365-2435.2006.01080.x

Kazakou, E., Violle, C., Roumet, C., Pintor, C., Gimenez, O., & Garnier, E. (2009). Litter quality and decomposability of species from a Mediterranean succession depend on leaf traits but not on nitrogen supply. Annals of Botany, 104, 1151–1161. doi: 10.1093/aob/mcp202

Lavorel, S., McIntyre, S., & Grigulis, K. (1999). Plant response to disturbance in a Mediterranean grassland: How many functional groups? Journal of Vegetation Science, 10, 661–672. doi: 10.2307/3237081

Lázaro-Nogal, A., Forner, A., Traveset, A., & Valladares, F. (2013). Contrasting water strategies of two Mediterranean shrubs of limited distribution: uncertain future under a drier climate. Tree Physiology, 33, 1284–1295. doi: 10.1093/treephys/tpt103

LeRoy, C. J., Wymore, A. S., Davis, R., & Marks, J. C. (2014). Indirect influences of a major drought on leaf litter quality and decomposition in a southwestern stream. Fundamental and Applied Limnology / Archiv für Hydrobiologie, 184(1), 1–10. doi: 10.1127/1863-9135/2014/0505

Lloret, F., Médail, F., Brundu, G., Camarda, I., Moragues, E., Rita, J., … Hulme, P. E. (2005). Species attributes and invasion success by alien plants on Mediterranean islands. Journal of Ecology, 93, 512–520. doi: 10.1111/j.1365-2745.2005.00979.x

Martin-StPaul, N. K., Limousin, J.-M., Vogt-Schilb, H., Rodríguez-Calcerrada, J., Rambal, S., Longepierre, D., & Misson, L. (2013). The temporal response to drought in a Mediterranean evergreen tree: comparing a regional precipitation gradient and a throughfall exclusion experiment. Global Change Biology, 19, 2413–2426. doi: 10.1111/gcb.12215

Paula, S., & Pausas, J. G. (2008). Burning seeds: germinative response to heat treatments in relation to resprouting ability. Journal of Ecology, 96, 543–552. doi: 10.1111/j.1365-2745.2008.01359.x

Pérez-Camacho, L., Rebollo, S., Hernández-Santana, V., García-Salgado, G., Pavón-García, J., & Gómez-Sal, A. (2012). Plant functional trait responses to interannual rainfall variability, summer drought and seasonal grazing in Mediterranean herbaceous communities. Functional Ecology, 26, 740–749. doi: 10.1111/j.1365-2435.2012.01967.x

Prieto, I., Roumet, C., Cardinael, R., Dupraz, C., Jourdan, C., Kim, J. H., … Stokes, A. (2015). Root functional parameters along a land-use gradient: evidence of a community-level economics spectrum. Journal of Ecology, 103, 361–373. doi: 10.1111/1365-2745.12351

Sans, F. X., Garcia-Serrano, H., & Afán, I. (2004). Life-history traits of alien and native senecio species in the Mediterranean region. Acta Oecologica, 26, 167–178. doi: 10.1016/j.actao.2004.04.001

Stanisci, A., Acosta, A. T. R., Di Iorio, A., & Vergalito, M. (2010). Leaf and root trait variability of alien and native species along Adriatic coastal dunes (Italy). Plant Biosystems, 144(1), 47–52. doi: 10.1080/11263500903454252

Targetti, S., Messeri, A., Staglianò, N., & Argenti, G. (2013). Leaf functional traits for the assessment of succession following management in semi-natural grasslands: a case study in the North Apennines, Italy. Applied Vegetation Science, 16, 325–332. doi: 10.1111/j.1654-109X.2012.01223.x

Valera-Burgos, J., Zunzunegui, M., & Díaz-Barradas, M. C. (2013). Do leaf traits and nitrogen supply affect decomposability rates of three Mediterranean species growing under different competition levels? Pedobiologia, 56, 113–119. doi: 10.1016/j.pedobi.2013.03.002

Vourlitis, G. L., Pasquini, S. C., & Mustard, R. (2009). Effects of Dry-Season N Input on the Productivity and N Storage of Mediterranean-Type Shrublands. Ecosystems, 12, 473–488. doi: 10.1007/s10021-009-9236-6

Werner, C., Zumkier, U., Beyschlag, W., & Máguas, C. (2010). High competitiveness of a resource demanding invasive acacia under low resource supply. Plant Ecology, 206, 83–96. doi: 10.1007/s11258-009-9625-0

Wolkovich, E. M., Lipson, D. A., Virginia, R. A., Cottingham, K. L., & Bolger, D. T. (2010). Grass invasion causes rapid increases in ecosystem carbon and nitrogen storage in a semiarid shrubland. Global Change Biology, 16, 1351–1365. doi: 10.1111/j.1365-2486.2009.02001.x


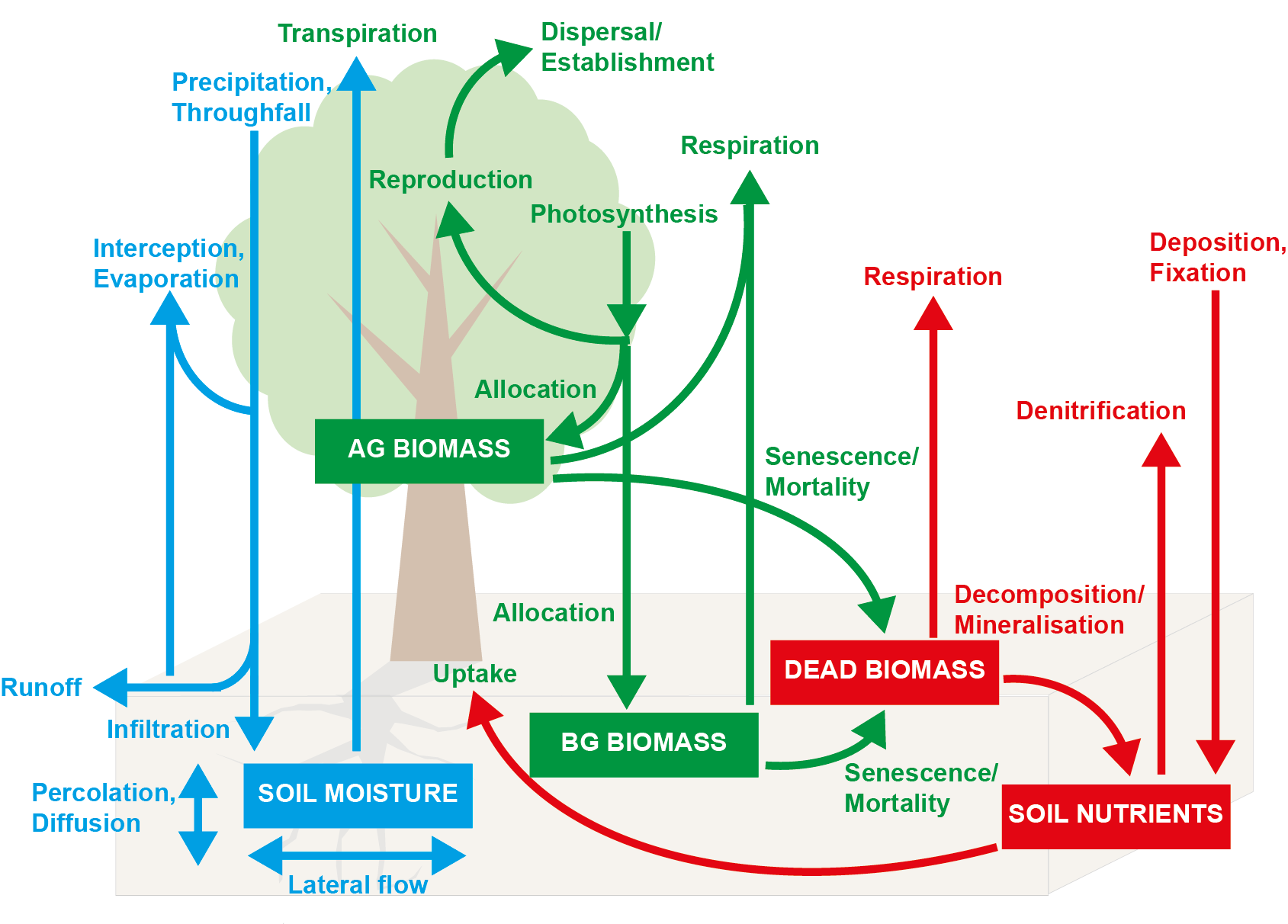


Figure S1. Overview of the stocks (boxes) and processes (arrows) of the developed model in the case study presented in the main text. Blue colours refer to the hydrological module, green colours to the vegetation module and red colours to the nitrogen module
